# The persulfidome of alveolar epithelial cells reveals persulfidation of calprotectin as critical to preserve its antifungal activity

**DOI:** 10.1016/j.isci.2026.117452

**Published:** 2026-09-06

**Authors:** Alba Río-Ropero, Álvaro González-Camuesco, Anna I. Mackey, Inês Caldeira, Mathieu Lepas, Edith Lucas-Vadillo, Cristina Cunha, Agostinho Carvalho, Matthew Z. Anderson, Jorge Amich

**Affiliations:** 1 Mycology Reference Laboratory (Laboratorio de Referencia e Investigación en Micología LRIM), National Centre for Microbiology, Instituto de Salud Carlos III (ISCIII), 28220 Madrid, Spain; 2 Department of Microbiology, College of Arts and Sciences, The Ohio State University, Columbus, OH 43210, USA; 3 Life and Health Sciences Research Institute (ICVS), School of Medicine, University of Minho, 4710-057 Braga, Portugal; 4 ICVS/3B’s – PT Government Associate Laboratory, 4710-057 Guimarães/Braga, Portugal; 5 Department of Microbial Infection and Immunity, School of Medicine, The Ohio State University, Columbus, OH 43210, USA; 6 Center for Genomic Science Innovation, University of Wisconsin-Madison, Madison, WI 53706, USA; 7 Laboratory of Genetics, University of Wisconsin-Madison, Madison, WI 53706, USA; 8 Manchester Fungal Infection Group (MFIG), Division of Evolution, Infection, and Genomics, Faculty of Biology, Medicine and Health, University of Manchester, Manchester M13 9NT, UK; 9 CiberInfec ISCIII, CIBER en Enfermedades Infecciosas, Instituto de Salud Carlos III, 28220 Madrid, Spain

**Keywords:** *Aspergillus fumigatus*, persulfidation, antifungal response, alveolar epithelial cells, persulfidome

## Abstract

Persulfidation is a post-translational modification involving the specific conversion of thiols (-SH) to persulfide groups (-SSH) on cysteine residues in target proteins. This modification plays critical roles in numerous physiological and cellular processes. Previously, we demonstrated that host cell persulfidation levels influence the effectiveness of antifungal immunity against the fungal pathogen *Aspergillus fumigatus*. In this study, we sought to further elucidate the relationship between persulfidation and the immune response. To this end, we defined the persulfidated proteins (the persulfidome) in alveolar epithelial cells that were challenged with *A. fumigatus* and/or exposed to a sulfide donor, which increases cellular persulfidation. Our findings suggest that persulfidation indirectly modulates immunity through changes in RNA metabolism and translation. Additionally, calprotectin was persulfidated in response to *A. fumigatus* exposure, and this modification is essential for maintaining its immune-related function under oxidative stress. Our results suggest that persulfidation positively influences generalized and specific immune responses.

## Highlights

• Multitude of proteins related to RNA metabolism are persulfidated in response to *A. fumigatus*
• Persulfidation of calprotectin is crucial for maintaining its core functions
• Reduced calprotectin persulfidation contributes to defective killing capacity in CTH^−/−^

## Graphical abstract

**Figure:**
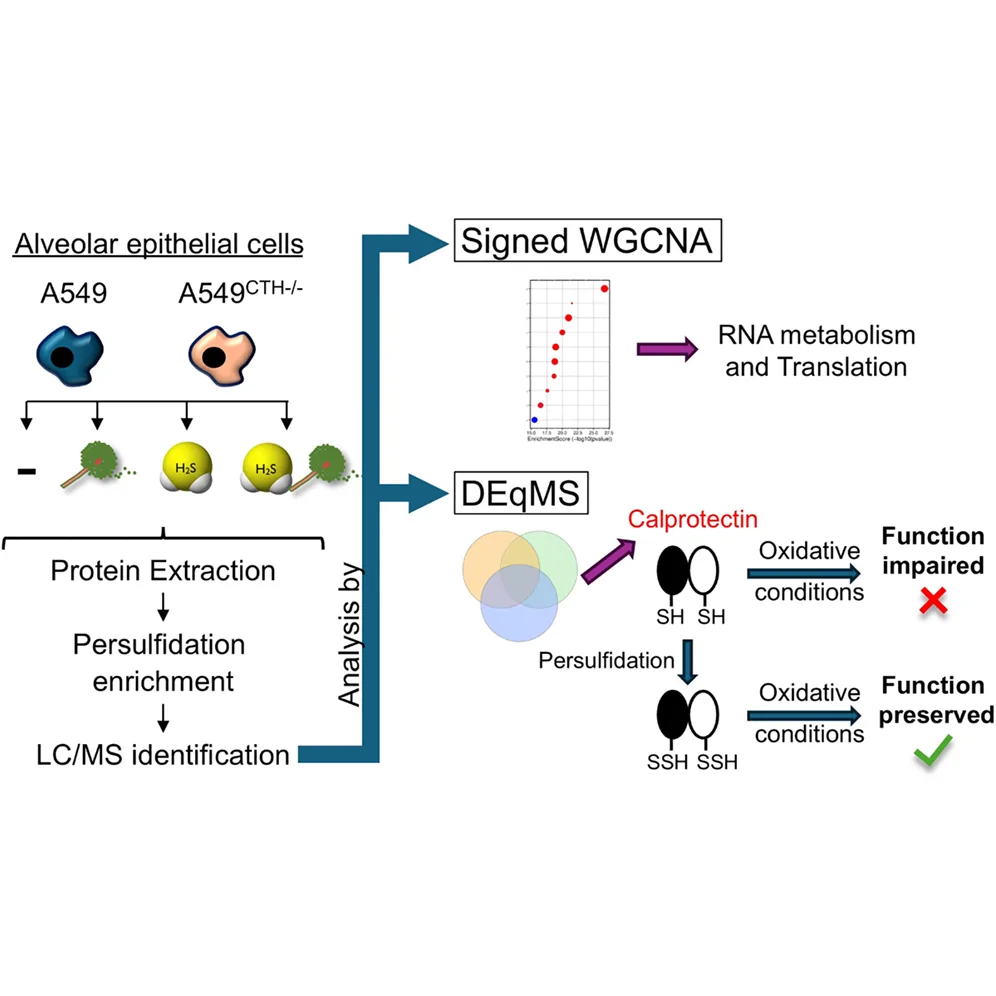

## Teaser

Immunology; Mycology; Proteomics

## Introduction

*Aspergillus fumigatus* is a filamentous saprophytic fungus found across the globe that produces thousands of airborne spores, also known as conidia, as part of its natural life cycle. Due to their high prevalence and small size (2–3 μm), conidia are swept into the air and are often inhaled by humans.^1^^,^^2^ Under normal circumstances, inhaled conidia are efficiently eliminated by the combined action of the respiratory epithelium and the innate immune response in the lungs.^3^ However, when the fungus encounters an individual with an imbalanced immune system, a range of disease states can result that are collectively termed aspergillosis.^3^^,^^4^ In immunocompromised individuals, *A. fumigatus* can cause invasive aspergillosis (IA), a lethal infection with a very high mortality rate, despite the availability of antifungal drugs.^5^ The increased prevalence of resistance to the front-line azole antifungal drugs adds to the potential impact of *A. fumigatus* on mortality rates.^6^^,^^7^^,^^8^

Immune function is a key prognostic predictor of IA infection and, accordingly, therapeutic interventions to enhance antifungal immunity are increasingly promising strategies.^9^^,^^10^ Neutropenic patients are particularly susceptible to fungal infection, including those caused by *A. fumigatus*, and patient morbidity can be mitigated via reconstitution of deficient immune lineages. In broader patient populations, susceptibility to IA can arise from immune imbalances caused by genetic polymorphisms,^11^ immunosuppressive agents,^12^^,^^13^^,^^14^ or even viral infections^15^ that impair the action of epithelial cells and immune effectors. In these cases, interventions that boost cellular immunity and improve the removal of invasive fungi may be sufficient to complement innate antifungal defenses.

Post-translational modifications are known to play important roles in the regulation of the innate immune response.^16^^,^^17^ Sulfide (H_2_S) is an important signaling molecule in humans,^18^^,^^19^ which is produced endogenously by at least three enzymes: cystathionine- β-synthase (CBS), cystathionine-γ-lyase (CTH) and 3-mercaptopyruvate sulfur-transferase (MST).^20^ Sulfide can promote pro-inflammatory and also anti-inflammatory responses^21^ through its numerous regulatory roles in immune cells.^22^ One of the main mechanisms through which H_2_S exerts its activity is protein persulfidation, a post-translational modification in which the thiol groups (-SH) of cysteine residues in target proteins are specifically modified to form a persulfide group (-SSH).^23^^,^^24^^,^^25^ This alteration can modulate protein activity and has a large impact on cell physiology and metabolism.^26^ Despite persulfidation being understudied due to its instability and chemical reactivity,^27^ its importance toward immunity is slowly being recognized.^28^^,^^29^^,^^30^

We previously reported that correct level of persulfidation of host proteins is important for the establishment of an effective immune response against *A. fumigatus*.^31^ Patients undergoing hematopoietic stem-cell transplantation who carry a variant in the cystathionine-γ-lyase encoding gene (*CTH*), which reduces its persulfidation activity in the lungs,^32^ exhibit an increased risk of developing IA. This loss of function of the *CTH* allele compromised the cellular antifungal response of the lungs, including abnormal phagocytosis, defective conidia killing, and dysregulated cytokine production.

Here, we show how persulfidation modulates cellular immunity to invasive *A*. *fumigatus*. Increasing the levels of persulfidation in alveolar epithelial cells with a sulfide donor enhanced the production of the chemoattractant cytokine interleukin (IL)-8. Furthermore, through a proteomic analysis, we identify persulfidated proteins that can influence the cellular immune response upon challenge with *A. fumigatus* conidia and/or a sulfide donor. Interestingly, we found that calprotectin subunits S100A8 and S100A9, which constitute an important antimicrobial and immunomodulatory molecule, are persulfidated. We demonstrate that persulfidation of calprotectin preserved its activity in the presence of oxidative stress, which can occur during the pro-inflammatory antifungal response, to maintain antifungal activity.

## Results and discussion

### A sulfide donor enhances the antifungal action of alveolar epithelial cells

**Figure 1:**
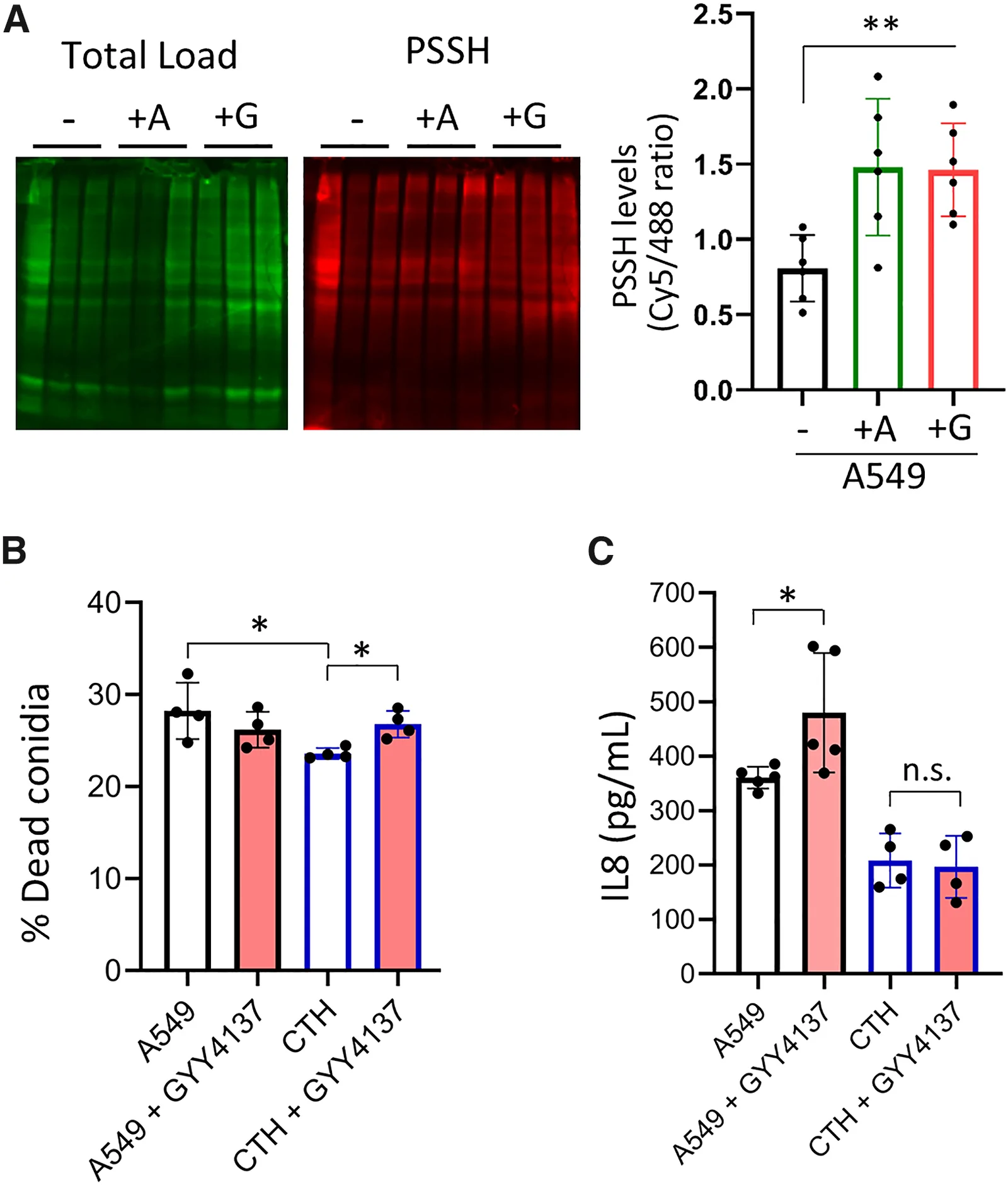
A sulfide donor has a positive effect on the cellular antifungal response (A) Persulfidation levels increased similarly in A549 cells when exposed to either *A. fumigatus* conidia (A) or the sulfide donor GYY4137 (G). (B) Incubation with 100 μM of the sulfide donor GYY4137 reconstituted the killing capacity of the CTH^−/−^ cells. However, it did not further increase the killing capacity of A549 cells. (C) GYY4137 augmented the IL-8 production in A549 cells upon challenge with *A. fumigatus*, but it did not have effect on IL-8 production of CTH^−/−^ cells. In all graphs, each datapoint represents a biological replicate and error bars represent standard deviation. All data were analyzed using one-way ANOVA with Tukey multiple comparisons. For all panels, significance is depicted: ∗*p* value < 0.05; ∗∗*p* value < 0.01; ∗∗∗*p* value < 0.001.

We previously showed that A549^CTH−/−^ cells (CTH^−/−^) have decreased persulfidation levels and a defective antifungal response,^31^ proving a role for endogenous CTH-derived H_2_S in regulating antifungal activity. Therefore, we hypothesized that the use of sulfide donors to increase persulfidation may boost the cellular antifungal response. Indeed, the sulfide donor GYY4137 increased persulfidation, mirroring the increment in cells that were exposed to *A. fumigatus* conidia (Figure 1A). To further test our hypothesis, we assayed the effect of the sulfide donor GYY4137 on the antifungal activity of A549 and CTH^−/−^ cells. Consistent with our prediction, addition of GYY4137 reconstituted the killing capacity of CTH^−/−^ cells (Figure 1B). In contrast to our expectation, GYY4137 did not enhance the killing capacity of parental A549 cells. However, it augmented IL-8 production in A549 cells, but not on CTH^−/−^ cells (Figure 1C). Therefore, addition of a sulfide donor increased cellular antifungal phenotypes differently in the presence and absence of CTH. This suggests that the sulfide donor likely exerts broad and indirect effects on the cell, making it challenging to link specific phenotypes to the role of CTH activity with the antifungal response.

**Figure 2:**
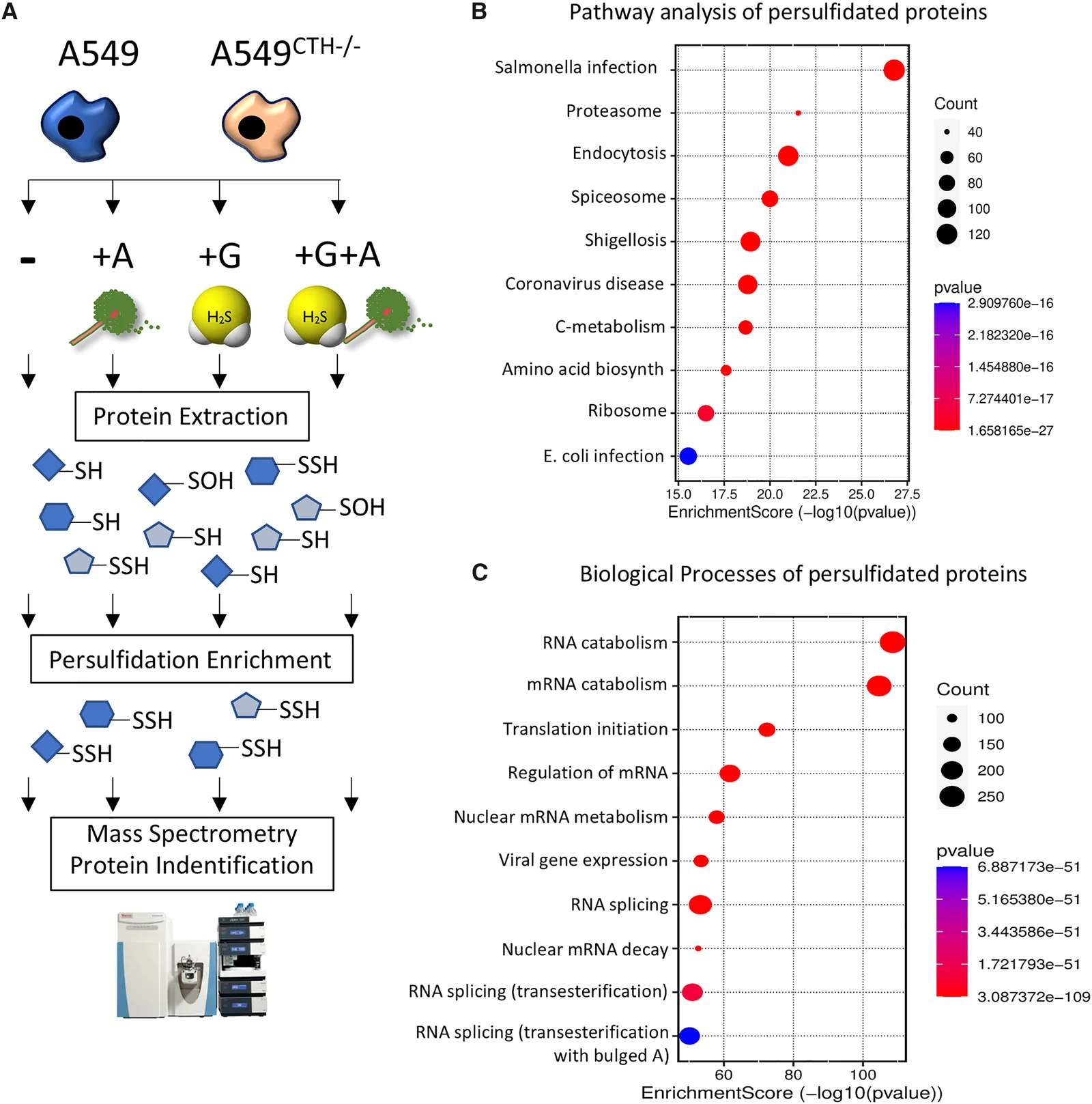
Differentially persulfidated proteins upon infection and sulfide donation suggest that persulfidation regulates RNA metabolism and responses to infection (A) Graphical representation of the process for production of the cellular persulfidomes based on treatment with *A. fumigatus* (+A) or the H_2_S donor (+G). (B) KEGG pathway enrichment analysis of all persulfidated proteins identified by liquid chromatography-mass spectrometry. Persulfidation associated with pathways involved in the responses to pathogens. (C) Gene Ontology analysis of enriched biological processes in all persulfidated proteins. Persulfidation strongly associated with RNA metabolism.

To better define the relationship between persulfidation and the cellular antifungal response, we analyzed the cellular persulfidome (proteome of persulfidated proteins) in A549 and in CTH^−/−^ cells upon challenge with *A. fumigatus* conidia and/or the sulfide donor GYY4137. All proteins were extracted, the persulfidated fraction was enriched using the dimedone-switch method^33^ following exposure to 100 μM of GYY4137, *A. fumigatus* conidia (multiplicity of infection 1:10), both, or neither stimulus (±GYY4137, ±*A. fumigatus)*, and the persulfidated proteins were identified by liquid chromatography-mass spectrometry (LC/MS) (Figure 2A). To understand processes generally regulated by persulfidation, we first examined all persulfidated proteins together, independent of the tested condition (Data S1). Persulfidated proteins were enriched in several Kyoto Encyclopedia of Genes and Genomes (KEGG) pathways related to infection, endocytosis, spliceosome and metabolism (Figure 2B). Enrichment analysis using Gene Ontology (GO) identified multiple biological processes associated with RNA metabolism (Figure 2C), suggesting that persulfidation may be involved in gene expression and RNA stability.

### Detection of modules of proteins that modify the persulfidation levels homogenously in certain conditions reveals potential candidates for a direct role in the antifungal response

**Figure 3:**
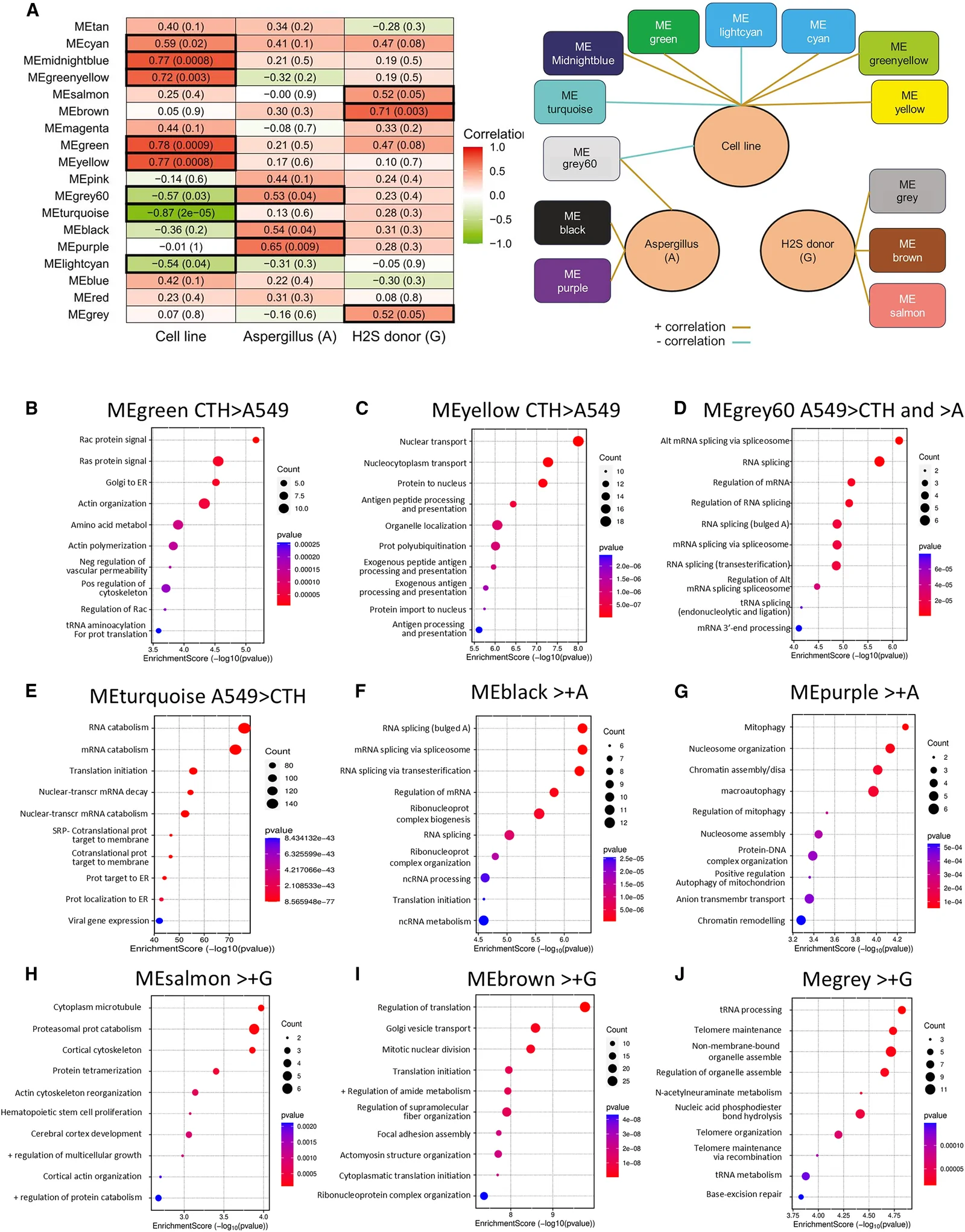
Signed weighted gene correlation network analysis of persulfidated proteins (A) Modules constructed from altered persulfidated protein levels across three conditions. The correlation coefficient is reported first, and the adjusted *p* value is indicated in parentheses for condition pair. (B) GO enrichment of biological processes in the MEgreen module: persulfidated proteins are more abundant in CTH^−/−^ cells than in A549 and also more abundant with the addition of the H_2_S donor. (C) GO enrichment of biological processes in the MEyellow module: persulfidated proteins are more abundant in CTH^−/−^ cells than in A549. (D) GO enrichment of biological processes in the MEgrey60 module: persulfidated proteins are more abundant in A549 than in CTH^−/−^ and also more abundant upon *Aspergillus* challenge. (E) GO enrichment of biological processes in the MEturquoise cluster: persulfidated proteins are more abundant in A549 than in CTH^−/−^. (F) GO enrichment of biological processes in the MEblack module: persulfidated proteins are more abundant upon *Aspergillus* challenge. (G) GO enrichment of biological processes in the MEpurple module: persulfidated proteins are more abundant upon *Aspergillus* challenge. (H) GO enrichment of biological processes in the MEsalmon module: persulfidated proteins are more abundant upon exposure to the H_2_S donor. (I) GO enrichment of biological processes in the MEbrown module: persulfidated proteins are more abundant upon exposure to the H_2_S donor. (J) GO enrichment of biological processes in the MEgrey module: persulfidated proteins are more abundant upon exposure to the H_2_S donor.

To identify protein modules associated with processes altered by persulfidation, we performed a signed Weighted Gene Co-expression Network Analysis (WGCNA) of samples across all four conditions (±GYY4137, ±*A. fumigatus*). Protein modules were considered to be significantly differentially persulfidated at an adjusted *p* value threshold of <0.05. Eight protein modules associated with differential persulfidation between A549 and CTH^−/−^ cells (Figure 3A). Three modules of persulfidated proteins were more abundant in A549 cells, and five modules were more abundant in CTH^−/−^ cells than in A549. Proteins in modules that are more abundant in A549 cells may be direct targets of persulfidation by CTH (MEgrey60 16 proteins, MEturquoise 1350 proteins, and MElightcyan 26 proteins, Figure 3A; Table S1). Interestingly, GO analysis of the MEgrey60 module showed significant enrichment for RNA processing and splicing (Figure 3D). The large MEturquoise cluster, which contains the vast majority of proteins dysregulated in CTH^−/−^ cells, was significantly enriched in RNA metabolism and translation (Figure 3E) and may indicate general metabolic dysregulation due to loss of persulfidation. In contrast, modules with increased persulfidation in CTH^−/−^ cells may reflect compensation by the other persulfidating enzymes (CBS and MST) to maintain homeostasis of specific cellular targets (MEcyan 32 proteins, MEmidnightblue 29 proteins, MEgreenyellow 44 proteins, MEgreen 133 proteins, and MEyellow 184 proteins, Figure 3A; Table S1). GO analysis revealed significantly enriched biological processes for only two of these modules: MEgreen, which was enriched for Rac- and Ras- signaling (Figure 3B), and MEyellow, which showed enrichment for nuclear transport (Figure 3C).

Next, we examined the modules of persulfidated proteins specifically associated with *Aspergillus* challenge (Figure 3A). All three significantly associated modules displayed increased persulfidation in the presence of conidia (MEgrey60 16 proteins, MEblack 97 proteins, and MEpurple 55 proteins). GO analysis detected enrichment of RNA-splicing processes in the MEblack module (Figure 3F) and of mitophagy, nucleosome organization, chromatin remodeling, and macroautophagy in the MEpurple module (Figure 3G).

Finally, we examined modules containing proteins with altered persulfidation upon exposition to the sulfide donor. All three significantly associated modules displayed increased persulfidation with GYY4137 (MEsalmon 33 proteins, MEbrown 269 proteins, and MEgrey 120 proteins). MEsalmon was enriched in microtubule and cytoskeletal organization, as well as proteasomal protein degradation (Figure 3H). MEbrown was enriched in several biological processes, including translation and cytoskeleton-related processes (Figure 3I). MEgrey was enriched in tRNA processing, telomere maintenance, and organelle assembly (Figure 3J).

Only one module was shared by more than one factor (MEgrey60, cell type and *A. fumigatus* challenge). Proteins in this module were less persulfidated in CTH^−/−^ cells than in A549 and increased in persulfidation upon challenge with conidia, suggesting that they might constitute a core response. As MEgrey60 is composed of only 16 proteins, we examined the relevance of each protein to a potential immune response. Both the RNA-splicing ligase RTCB and the RNA helicase DDX5 have been reported to regulate interferon production,^34^^,^^35^ and the mRNA processing factor SRSF7 is required for optimal expression of interferon-stimulated genes in macrophages.^36^ The class I myosin MYO1E triggers macrophage spreading and MHC class II presentation in macrophages^37^ and neutrophil extravasation.^38^ The RabGTPase RAB14 was reported to regulate maturation of phagosomes containing *Candida albicans*.^39^ The protein deacetylase SIRT6 promotes macrophage M2 polarization^40^ and has been proposed as a master regulator of immune cell metabolism.^41^ Therefore, several proteins in this cluster may have a direct effect on the cellular anti-*Aspergillus* response and are direct targets of persulfidation by CTH. No modules were significantly associated with exposure to *Aspergillus* and the sulfide donor, suggesting that protein persulfidation dynamics were condition-specific, having a minimal overlap between these conditions. This could explain the inconsistency in increased conidia killing by addition of GYY4137 between cell lines (Figure 1A).

Proteins unable to be consistently identified by mass spectrometry across conditions presented challenges in analysis but suggest differential persulfidation of particular proteins between conditions. For example, some proteins were detected in A549 but absent from CTH^−/−^ cells and may account for intrinsic defects of CTH^−/−^ cells in mounting an antifungal response (Table S2). At least two of those proteins may be directly responsible for the defective antifungal response of CTH^−/−^ cells: RFX1, a transcriptional regulator of MHC class II,^42^^,^^43^ and AP2A1, an adaptor protein that promotes acetylation of α-tubulin, fundamental for inflammasome activation.^44^

### Detection of proteins that change the persulfidation levels upon challenge identifies calprotectin as a candidate link with the immune response

Next, to specifically define proteins with altered persulfidation upon challenge with *Aspergillus* and/or exposure to the H_2_S donor, we performed differential abundance analysis by DEqMS.^45^ When we compared the abundance of persulfidated proteins in A549 without *A. fumigatus* challenge (-G-A) with challenge with *Aspergillus* (-G+A), we detected only four proteins that were significantly less abundant in A549-G+A (>2-fold change, *p* value < 0.05, false discovery rate [FDR]<0.15) and no proteins with increased abundance (Data S2). These four proteins, ESD, PPP2R2D, PWP1, RTF2, have no obvious relationship to cellular immunity in response to *A. fumigatus*. Six persulfidated proteins were significantly less abundant in A549-G-A cells vs. A549+G+A cells, four of which were identical to those in the A549-G±A comparison. The A549-G-A vs. A549+G+A comparison also found five proteins that were more abundant (i.e., increased persulfidation levels) in the presence of *Aspergillus* and a sulfide donor compared with the absence of both (Data S3). These differentially persulfidated proteins could be involved in enhancing the cellular antifungal response, but manual inspection of the proteins did not reveal any obvious relationships with the immune response, suggesting again that many effects of persulfidation on the immune system are likely indirect. For instance, FAM98B, which is more abundant in A549-G-A vs. A549+G+A, is known to positively stimulate the arginine methylase PRMT1,^46^ which influences the immune system and inflammation^47^ and also activates the kinase TBK1^48^ that is important in immune sensing.^49^

**Figure 4:**
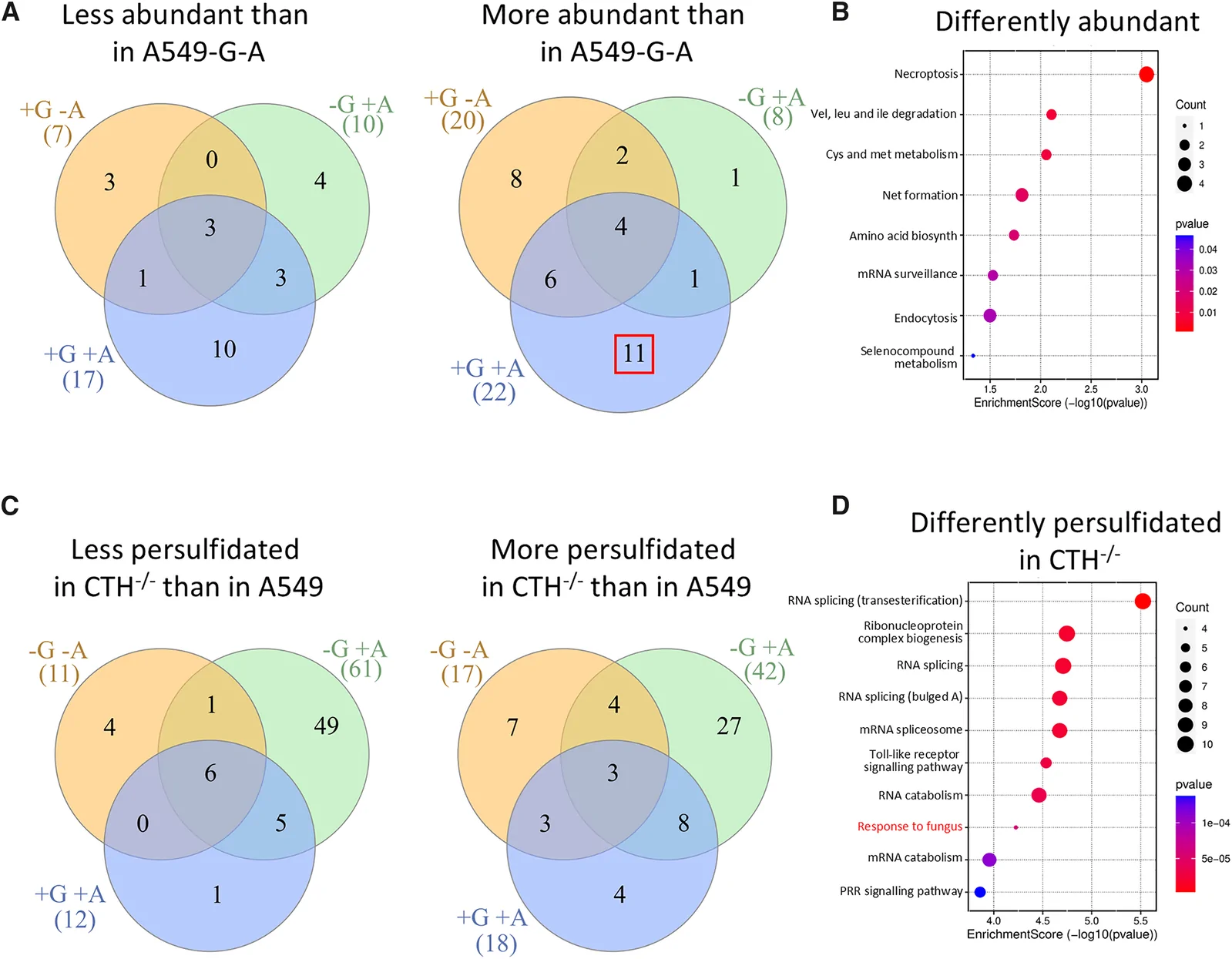
DEqMS analysis of differentially persulfidated proteins (A) Persulfidated proteins with decreased or increased abundance in the A549 cells compared with the cells exposed to *Aspergillus* (+A), GYY4137 (+G), or both (+G+A). (B) Proteins identified in (A) were enriched in pathways detected related to necroptosis, amino acid metabolism, and neutrophil response by KEGG analysis. (C) Persulfidated proteins with decreased or increased abundance in the CTH^−/−^ cells compared with the A549 cells in each of the indicated conditions. (D) Gene Ontology analysis of proteins in (C) detected a significant enrichment in biological processes related to RNA splicing, Toll-like receptor signaling, and response to fungus. The Venn diagrams were generated using the free InteractiVenn online platform^50^ (https://www.interactivenn.net/).

The low number of differentially abundant persulfidated proteins with clear connections to the immune response led us to expand the scope of the investigation by removing the requirement for an FDR<0.15 and attending only to the *p* value < 0.01 and the >2-fold change thresholds. The less stringent analysis detected 30, 15, and 39 proteins with altered abundance in A549-G+A, A549+G-A, and A549+G+A conditions, respectively, when compared with A549-G-A (Figure 4A; Table 1, Data S2, S3, and S4). Taken together, these 84 proteins were enriched for necroptosis, amino acid metabolism, and neutrophil response by KEGG pathway analysis (Figure 4B). Interestingly, STAT6 was less persulfidated upon double (+G+A) but not single (-G+A or +G-A) treatment of A549 cells compared with the absence of any stimulus (Table 1). STAT6 promotes Th2 signaling and has been associated with airway hyperresponsiveness upon *Aspergillus* exposure.^51^ Reduced persulfidation might modulate STAT6 activity and contribute to a balanced immune response. Additionally, NCCRP1 had the greatest increase in persulfidation upon *Aspergillus* challenge (both alone and in combination with the sulfide donor, Table 1). Prior work has described NCCRP1 as a receptor of non-specific cytotoxic cells in fish^52^ that are important for the antifungal immune response.^53^

**Table 1:**
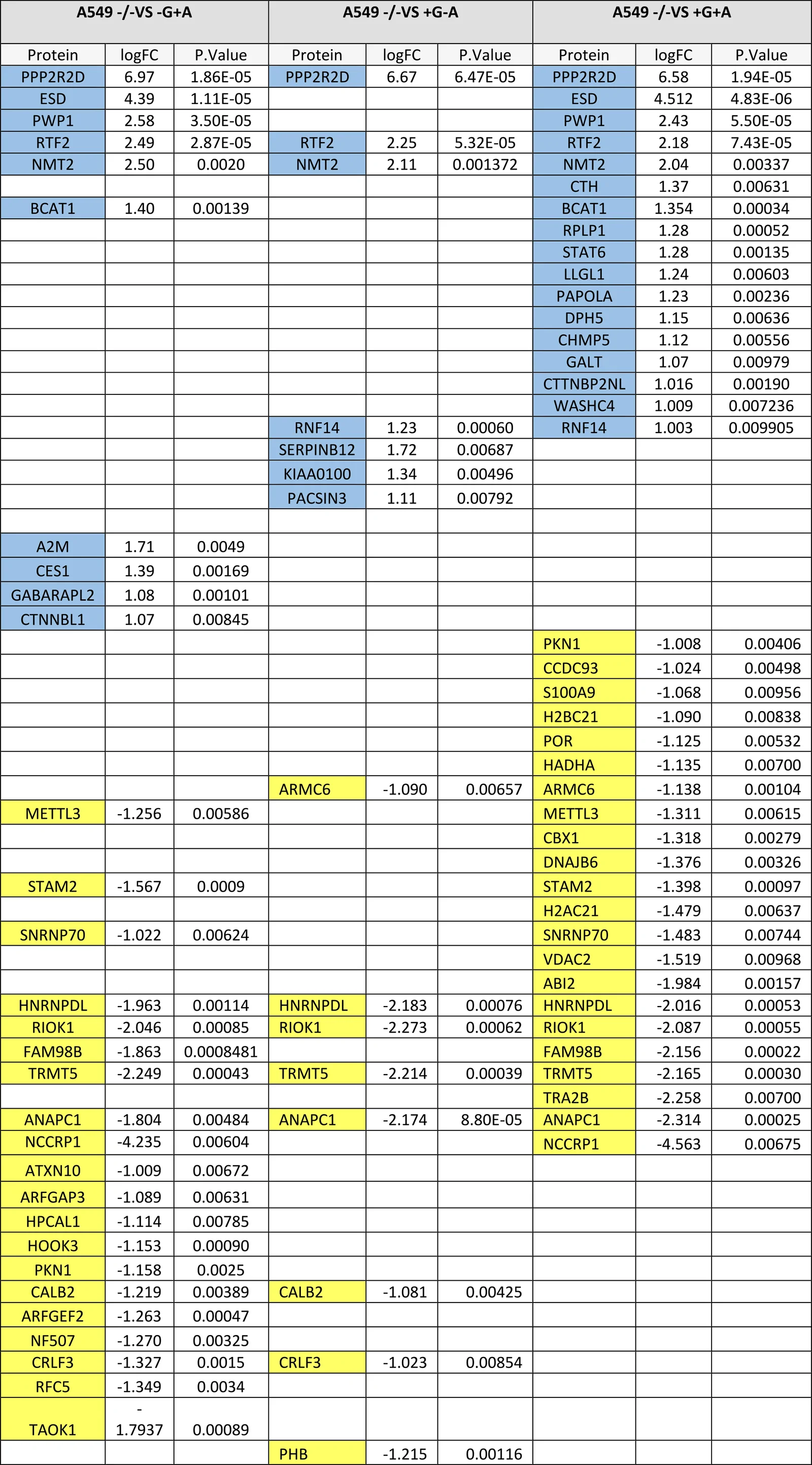
Persulfidated proteins the abundance of which decreases (blue) or increases (yellow) in A549 cells upon challenge with *A. fumigatus* conidia (A) and/or the sulfide donor (G)

Among the differentially abundant persulfidated proteins, persulfidation levels of 11 proteins increased specifically upon double (+G+A) but not single (-G+A or +G-A) treatment (Figure 4A; Table 1), suggesting that they might be important for the increased IL-8 production that contributes to an improved antifungal response. Interestingly, S100A9, a calcium-binding protein that forms calprotectin when it dimerizes with S100A8, was one of this more persulfidated proteins upon double challenge (Table 1). Calprotectin is critical in combatting infection via leukocyte recruitment, stimulation of cytokine production, and direct antimicrobial function,^54^ including anti-*Aspergillus* activity.^55^^,^^56^ Production of S100A9 by airway epithelial cells has been shown to potentiate macrophage function,^57^ in part, by increasing the production of MUC5AC.^58^ Interestingly, production of oxidative radicals during an antimicrobial response oxidates calprotectin cysteine residues, creating a disulfide cross-link that alters its structure and increases its proteolytic degradation.^59^

**Figure 5:**
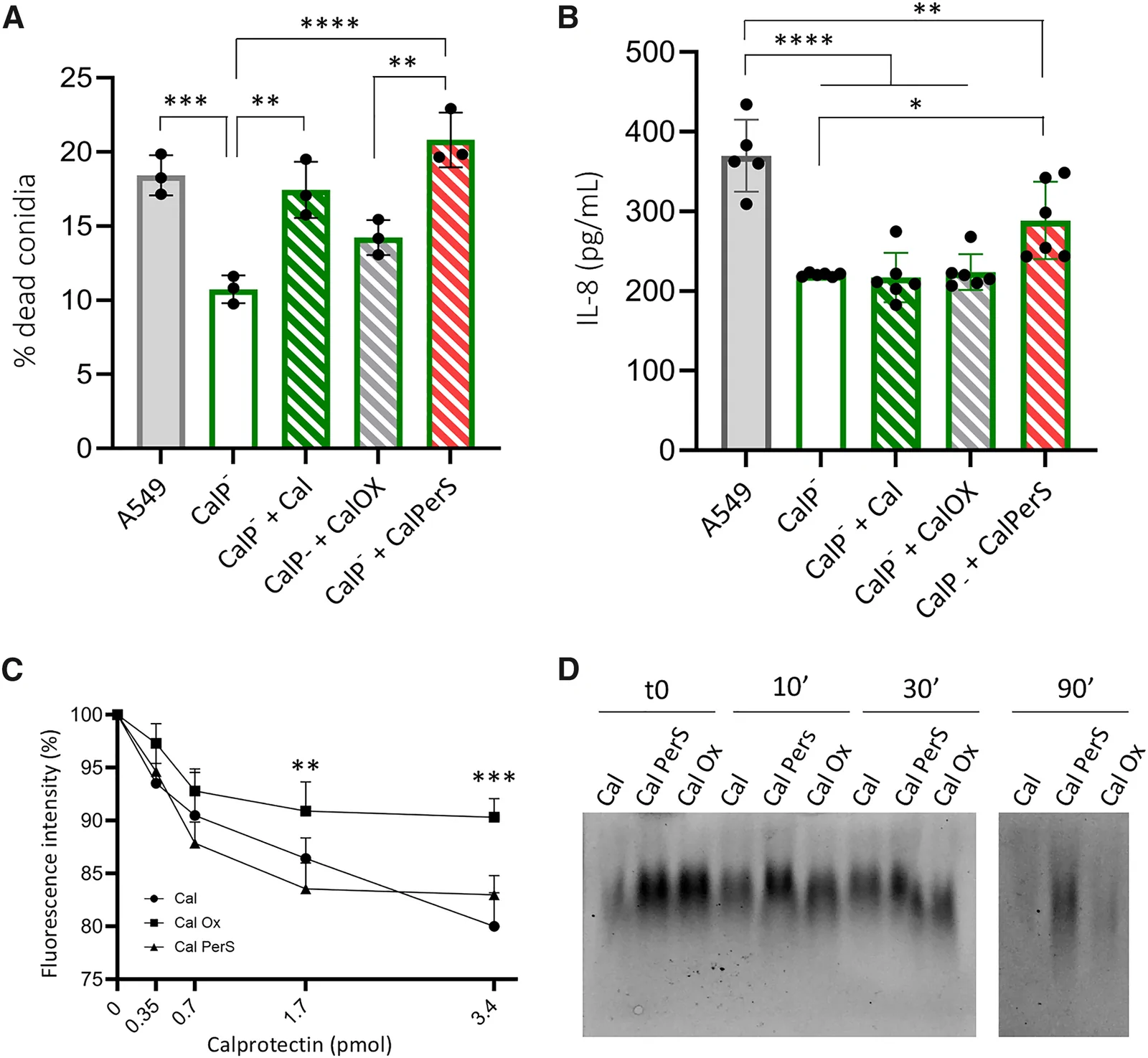
Persulfidation of calprotectin preserves its function in the presence of oxidative stress (A) Deletion of S100A9 in A549 (calprotectin negative, CalP^−^) significantly reduced the conidia killing capacity of the cells. Addition of recombinant calprotectin reconstituted killing to wild-type levels. However, if calprotectin had been previously hyperoxidated *in vitro*, it failed to fully reconstitute CalP^−^ killing action. Persulfidation of calprotectin *in vitro* prior to the oxidation treatment preserved its function, maintaining its capacity to reconstitute killing. (B) Addition of untreated or hyperoxidated recombinant calprotectin to CalP^−^ cells did not affect IL-8 production upon *A. fumigatus* challenge. Addition of persulfidated calprotectin significantly augmented CalP^−^ IL-8 production but not to wild-type levels. (C) Oxidated calprotectin had a significantly decreased capacity to reduce the fluorescence intensity produced by the activity of the DNAzyme 17E, reflecting a reduced Zn^2+^ chelation action. Previous persulfidation protected calprotectin chelation activity. (D) Trypsin proteolysis assay. Oxidized calprotectin was digested faster than the untreated and previously persulfidated proteins. In (A) and (B), each datapoint represents a biological replicate and error bars represent standard deviation. Data were analyzed using one-way ANOVA with Tukey multiple comparisons. In (C), error bars represent starndard deviation of three independent replicates. Data were analyzed using an unpaired, two-tailed Student *t* test. For all panels significance is depicted: ∗*p* value < 0.05; ∗∗*p* value < 0.01; ∗∗∗*p* value < 0.001.

We hypothesized that persulfidation of calprotectin might prevent its degradation and preserve its activity in the presence of oxidative stress. To test this hypothesis, we constructed an A549^S100A9−/-^ (CalP-) cell line and confirmed that these cells have a defect in conidia killing (Figure 5A) and IL-8 production (Figure 5B). Addition of recombinant calprotectin reconstituted CalP- killing capacity (Figure 5A) but not IL-8 production (Figure 5B). However, if calprotectin had been previously hyperoxidated *in vitro*, it failed to reconstitute the cells’ killing capacity and demonstrated that oxidation impairs calprotectin function (Figure 5A). In agreement with our hypothesis, if calprotectin had been persulfidated *in vitro* before the hyperoxidative treatment, it retained the capacity to wild-type levels of killing conidia (Figure 5A). Moreover, IL-8 production significantly improved with addition of persulfidated calprotectin that either untreated or hyperoxidated calprotectin failed to do (Figure 5B). These effects indicate that persulfidation of calprotectin preserves its function in the presence of oxidative conditions. To further confirm this observation, we examined the role of persulfidation on two calprotectin core functions: Zn^2+^ chelation and resistance to proteolysis.

As the chelating properties of calprotectin were already shown to be reduced by oxidation,^60^ we firstly examined if persulfidation can protect this activity. We measured the Zn^2+^ chelating capacity using a recently published methodology,^61^ which employs a Zn^2+^ dependent DNAzyme (17E) to cleave an oligonucleotide that contains a fluorophore in its 3′ end and a quencher in its 5′ end. Therefore, the DNAzyme activity liberates the fluorophore and triggers fluorescence emission. In the presence of functional calprotectin, the Zn^2+^ cofactor is chelated, thus the DNAzyme activity is blocked and the emission of fluorescence reduced (Figure 5C). When calprotectin had been oxidated, fluorescence emission was significantly higher (Figure 5C), reflecting a decrease in calprotectin chelation capacity. In contrast, if calprotectin had been persulfidated prior to the hyperoxidative treatment, it maintained its Zn^2+^-chelating capacity (Figure 5C). Besides, it has also been shown that oxidation of calprotectin increases its susceptibility to proteolysis.^59^ Therefore, we measured if persulfidation could also protect calprotectin from this negative effect of oxidation. Indeed, we found that, although oxidation makes calprotectin more vulnerable to trypsin digestion, previous persulfidation prevented this enhanced susceptibility (Figure 5D). Hence, it can be concluded that persulfidation protects calprotectin activity under oxidative conditions.

**Figure 6:**
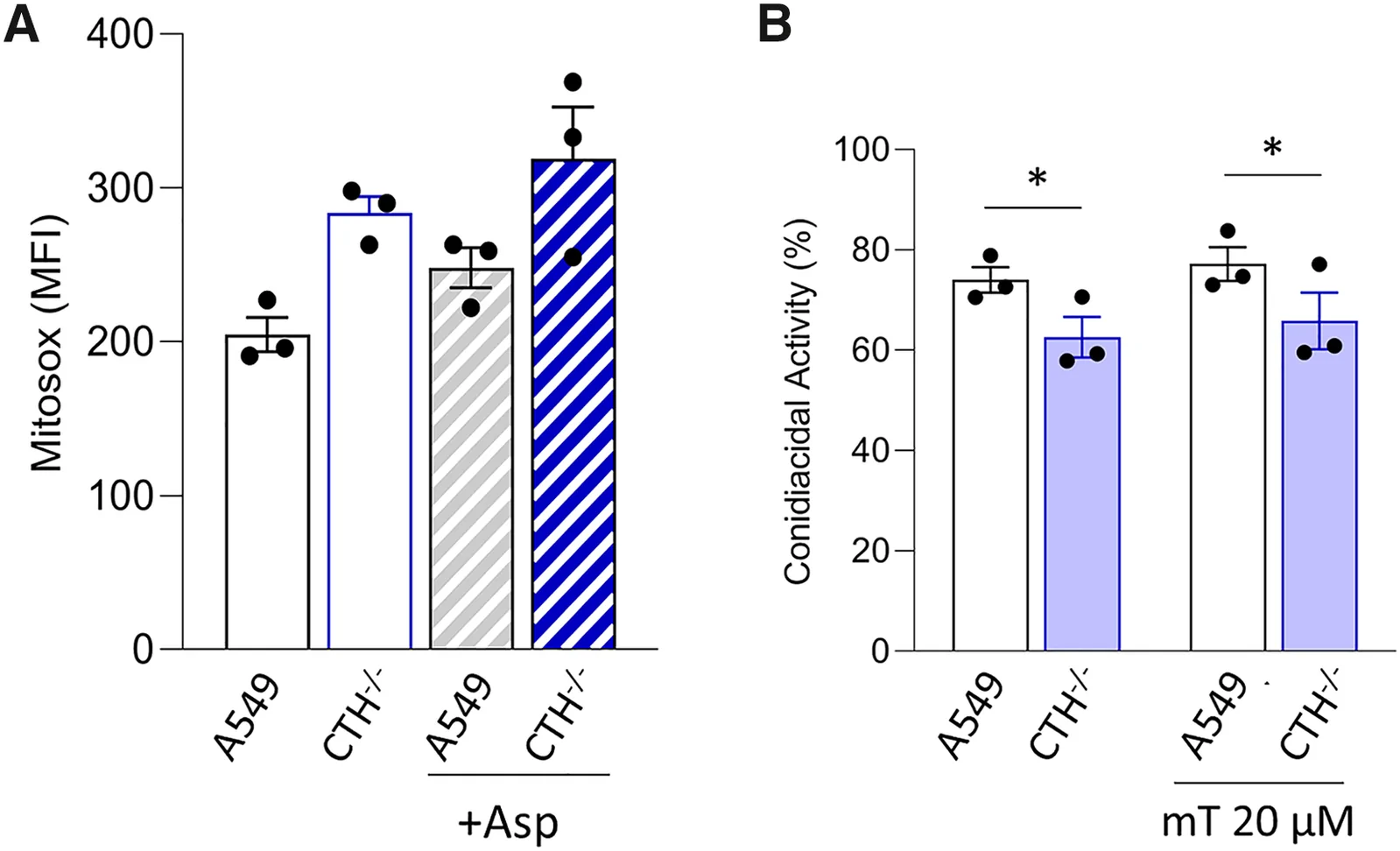
Persulfidation is implicated in mitoROS production, but this does not drive the defect in fungal killing in the absence of CTH (A) Quantification of mean fluorescence intensity (MFI) for the mitoROS levels in three independent replicates. (B) The CTH^−/−^ defect in conidiacidal activity is not reconstituted by the mitoROS scavenger mitoTEMPO (mT). Data were analyzed with an unpaired two-tailed Student *t* test. The experiment was repeated three independent times, error bars represent standard deviation. For all panels significance is depicted: ∗*p* value < 0.05; ∗∗*p* value < 0.01; ∗∗∗*p* value < 0.001.

Additionally, we noted that three of the eleven proteins with increased persulfidation were related to mitochondria: POR (NADPH-cytochrome P450 reductase), VDAC-2 (voltage-dependent anion-selective channel protein 2), and HADHA (hydroxyacyl-CoA dehydrogenase trifunctional multienzyme complex), suggesting a potential role of persulfidation in mitochondria during infection. As previously described,^62^ challenge with *Aspergillus* caused a modest increase in mitochondrial ROS (mitoROS) (Figure 6A). Instead, levels of mitoROS were significantly higher in CTH^−/−^ cells both in steady state conditions and upon fungal challenge (Figure 6A). This agrees with previous knowledge, as loss of CTH was shown to cause increased levels of mitoROS in endothelial cells.^63^ However, treatment of CTH^−/−^ cells with the mitoROS scavenger mitoTEMPO did not reconstitute their killing defect (Figure 6B), suggesting that the increase in mitoROS is not a primary driver of the impaired antifungal activity in the absence of CTH.

### Persulfidation of calprotectin partly accounts for the defective response of the CTH^−/−^ cell line

Although we had proved a role for persulfidation in calprotectin function, a connection to the defective antifungal response of the CTH^−/−^ cell line remained unresolved. To identify proteins with differential persulfidation, we compared A549 and CTH^−/−^ cells in the absence and presence of GYY4137 and *A. fumigatus* (Figure 4C and Data S5). In the absence of challenge (A549-G-A vs. CTH^−/−^-G-A), we found 17 and 11 proteins with increased and reduced persulfidation in CTH^−/−^ cells, respectively (>2-fold change, FDR<0.05). These protein alterations likely reflect basal defects of persulfidation in the absence of CTH. Importantly, there were no differences in protein persulfidation in the A549+G-A vs. CTH^−/−^+G-A comparison, demonstrating that addition of the sulfide donor restored normal persulfidation levels of cellular proteins (Data S5). In contrast, *Aspergillus* exposure (A549-G+A vs. CTH^−/−^-G+A) exacerbated differences in protein persulfidation between wild-type and CTH^−/−^ A549 cells; 60 and 42 proteins displayed decreased and increased persulfidation, respectively, in CTH^−/−^ cells (Data S5). This suggests that CTH actively modulates persulfidation upon fungal challenge. Finally, exposure to both *A. fumigatus* and the sulfide donor GYY4137 (A549 + G+A vs. CTH^−/−^+G+A) altered persulfidation of 30 total proteins, 12 with reduced and 18 increased persulfidation in CTH^−/−^ (Data S5). The reduced number of altered proteins in this comparison vs. *Aspergillus* exposure alone further supports the contributions of GY4137 to restored persulfidation.

**Figure 7:**
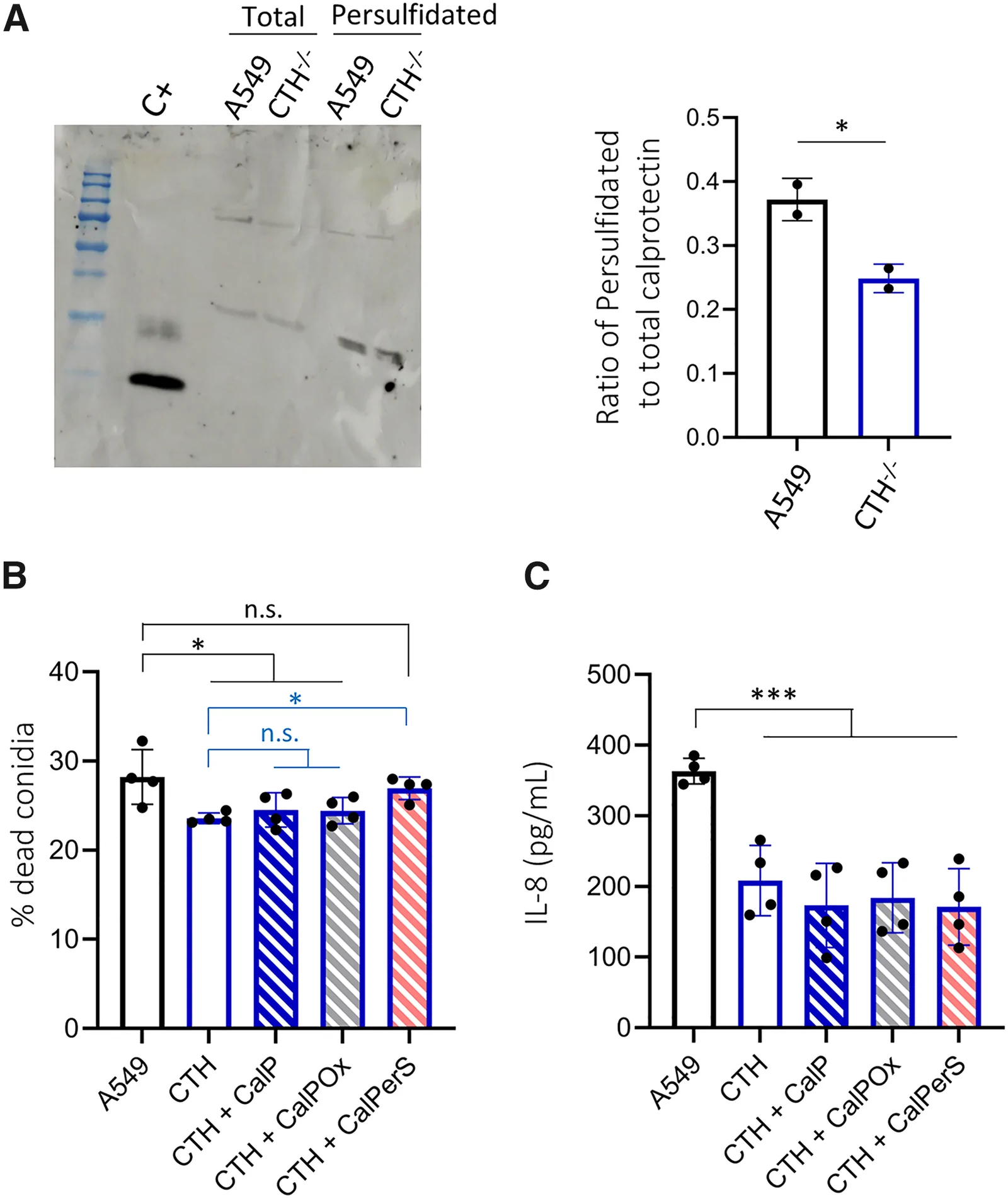
Persulfidated calprotectin partially reconstitutes the killing capacity of CTH^−/−^ cells (A) The CTH^−/−^ cell line contains a decreased ratio of persulfidated calprotectin compared with the A549 cell line. (B) Addition of untreated or hyperoxidated recombinant calprotectin to CTH ^−/−^ cells did not improve their conidia killing capacity. Addition of persulfidated calprotectin partially reconstituted CTH ^−/−^ killing ability. The killing capacity of A549 was compared with all CTH^−/−^ conditions using one-way ANOVA with Dunnet multiple comparisons (black). The killing capacity of CTH^−/−^ was compared with the three CalP supplemented CTH^−/−^ conditions using one-way ANOVA with Dunnet multiple comparisons (blue). (C) Calprotectin did not influence IL-8 production in CTH^−/−^ cells upon *A. fumigatus* challenge. In all graphs, each datapoint represents a biological replicate and error bars represent standard deviation. Data were analyzed using one-way ANOVA with Tukey multiple comparisons. For all panels, significance is depicted: ∗*p* value < 0.05; ∗∗*p* value < 0.01; ∗∗∗*p* value < 0.001.

We screened for persulfidated proteins with altered abundances in CTH cells upon exposure to *A. fumigatus*, which recovered A549 level upon GY4137 exposure. This comparison identified 26 and 49 proteins with decreased and increased persulfidation, respectively (Figure 4C and Data S5). GO analysis detected a significant enrichment for processes related to RNA splicing, Toll-like receptor signaling, and “response to fungus” among these proteins (Figure 4D), which suggested a direct link to the defective anti-*Aspergillus* response of CTH^−/−^ cells. The four identified proteins that belong to this GO biological term (GO:0009620) were LTF (lactoferrin, an iron-binding protein important in nutritional immunity and which shows antifungal activity^64^), DCD (dermcidin, an antimicrobial peptide with antifungal activity^65^), BCL-10 (involved in NF-κB activation^66^), and S100A8 (one of the heterodimer calprotectin subunits). We confirmed that, indeed CTH^−/−^ cells contain reduced levels of persulfidated calprotectin (Figure 7A). Thus, we hypothesized that the defective antifungal response in CTH^−/−^ cells might be partially due to the reduced persulfidation of calprotectin. According to this postulate, addition of *in vitro* persulfidated calprotectin significantly reconstituted the killing capacity of CTH^−/−^ cells (Figure 7B), although it did not restore IL-8 levels (Figure 7C).

Taken together, our results indicate that the impact of persulfidation on the cellular antifungal response is primarily indirect, likely mediated through its influence on cytoskeletal dynamics, cellular energetics, RNA metabolism, and protein translation. Notably, persulfidation of proteins appears to play a greater role in orchestrating an effective immune response than in directly killing *A. fumigatus*—a conclusion supported by both our prior findings^31^ and the observed effects of GYY4137 on the cellular antifungal capacity (Figure 1). Yet, persulfidation of the important antimicrobial peptide calprotectin preserved its function under oxidative conditions that typically lead to its turnover. High levels of oxidizing agents produced during infection by neutrophils—and likely other phagocytes—that typically degrade calprotectin and impair its activity may be buffered by persulfidation.^59^^,^^60^ Therefore, persulfidation of calprotectin seems to constitute a direct link with the efficient clearance of *A. fumigatus*. Given the complexities of direct and indirect targets of persulfidation that may contribute to cellular antifungal response, *in vivo* studies examining the integrated immune response will be essential to fully elucidate these mechanisms.

### Limitations of the study

A limitation worth noting in this study is that we did not normalize persulfidation to the total protein abundance. We acknowledge that this precludes us from definitively distinguishing whether changes in the abundance of persulfidated proteins reflect altered stoichiometry or parallel changes in total protein expression. Nevertheless, given the known changes in persulfidation stoichiometry in CTH cells (as we showed in Sueiro-Olivares et al.^31^) and upon *Aspergillus* or sulfur donor exposure (Figure 1A), we believe that the majority of detected proteins reflect true variations in persulfidation levels. Critically, the biological interpretations remain valid: any increase or decrease in the absolute levels of a persulfidated protein (whether driven by altered protein expression or persulfidation stoichiometry) carries functional consequences for the relevant pathways.

## Resource availability

### Lead contact

Requests for further information and resources should be directed to and will be fulfilled by the lead contact, Jorge Amich (jamich@isciii.es).

### Materials availability

• The cell lines generated in this study are commercially available via Editgene (Cat. no.: EDC07545 and EDC90108).
• This study did not generate new unique reagents.

### Data and code availability

• The raw proteomics data have been deposited at PRIDE as Project PXD076106 and are publicly available as of the date of publication.
• This paper does not report original code.
• Any additional information required to reanalyze the data reported in this paper is available from the lead contact upon request.

## Acknowledgments

We acknowledge the use of the Biology Mass Spectrometry Facility (RRID code SCR_020987) of the Faculty of Medicine, Biology and Health at the University of Manchester. Particularly, we would like to thank Ronan O’cualain for help with the S-trap procedure, David Knight for guidance with the design of the proteome experiment, and Julian Selley for the analysis and normalization of the raw data. We would like to thank the help and support of colleagues at the Manchester Infection Group (MFIG) and the Spanish Mycology Reference Centre (LRIM). This work was funded by an MRC Career Development Award (MR/N008707/1) and the Proyecto de Generación del Conocimiento (10.13039/501100011033Agencia Estatal de Investigación, PID2022-136343OA-I00). A.I.M. and M.Z.A. were funded by 10.13039/100000002NIH R01 AI148788. Additional support was provided by the 10.13039/501100001871Fundação para a Ciência e a Tecnologia (FCT) (UID/06304/2023, LA/P/0050/2020, 2022.06674.PTDC, PMTargets/0003/2024, and 2023.02637.BD), the “la Caixa” Foundation and FCT under the agreement LCF/PR/HR24/52440014, the “10.13039/100010434la Caixa” Foundation under the agreement LCF/PR/HR22/52420003, and the EU Horizon 2020 research and innovation programme under grant agreement no 847507. This work was also supported by the 10.13039/100000002National Institutes of Health grant 1R01AI148788 to M.Z.A.

## Author contributions

A.R.-R., investigation, data curation, formal analysis, and visualization; A.G.-C., investigation, data curation, and formal analysis; A.I.M., investigation and methodology; M.L., investigation and visualization; I.C., investigation and visualization; E.L.-V., investigation; C.C., conceptualization, resources, supervision, validation, and writing – review and editing; A.C., conceptualization, resources, supervision, validation, writing – review and editing, and funding acquisition; M.Z.A., conceptualization, software, data curation, formal analysis, supervision, validation, writing – review and editing, and funding acquisition; J.A., conceptualization, data curation, formal analysis, funding acquisition, methodology, project administration, supervision, validation, visualization, and writing – original draft.

## Declaration of interests

The authors declare no competing interests.

## STAR★Methods

### Key resources table

**Table:** 

REAGENT or RESOURCE	SOURCE	IDENTIFIER
**Antibodies**		

Calprotectin Monoclonal Antibody (MAC387)	Invitrogen	Cat# MA1-81381; RRID: AB_2184262
Goat anti-mouse IgG (H + L)	Invitrogen	Cat#G21040; RRID: AB_2536527

**Fungal strains**		

*Aspergillus fumigatus* wild-type	Hearn and Mackenzie^67^	ATCC 46645
*Aspergillus fumigatus AfS148 [*^*p*^*gpdA::gfp2-5*	Lother et al.^68^	N/A

**Chemicals, peptides, and recombinant proteins**		

Dulbecco’s Modified Eagle Medium (DMEM)	Sigma-Aldrich	Catalog# D6429
Fetal Bovine Serum	Sigma-Aldrich	Catalog# F7524
Penicillin-Streptomycin	Sigma-Aldrich	Catalog# P4333
Recombinant Human S100A8/S100A9 Heterodimer	R&D Systems	Catalog# 8226-S8
DL-Ditiotreitol (DTT)	Sigma-Aldrich	Catalog# D0632
4-(2-hydroxyethyl)-1-piperazine-ethanesulfonic acid (HEPES)	Sigma-Aldrich	Catalog# H3375
Potassium (poly)-sulfide		Catalog# 12665
Sodium hypochlorite >14% active Chlorine	Fluorochem	Catalog# F545154
GYY4137 (dichloromethane-fre)	Cymit-Biosynth	Catalog# 3D-AED14921
Propidium Iodine	Sigma	Catalog# 537059
RIPA buffer	Thermo-Fisher Scientific	Catalog# 89901
4-Chloro-7-nitrobenzofurazan (ClNBF)	Sigma-Aldrich	Catalog# 25455
Neocuproine	Sigma-Aldrich	Catalog# 1029680005
Dimedone azide-2 (DAz-2)	Cayman Chemical	Catalog# 13382
Cy5 alkyne	Lumiprobe	Catalog# B30B0
TBTA-Cu (II) complex	Lumiprobe	Catalog# 21050
DCP-BIO1	Merck-Millipore	Catalog# NS1226
Trypsin	Promega	Catalog# V5111
Dynabeads™ Streptavidin Magnetic Beads	Thermo-Fisher Scientific	Catalog# 11205D
Lumitein Protein gel stain	Biotium	Catalog# BT-21002–1
Calcofluor White	Sigma-Aldrich	Catalog# F3543

**Critical commercial assays**		

Desalting Zeba, MWCO de 7K	Thermo-Fisher Scientific	Catalog# 89882
ELISA MAX™ Deluxe Set Human IL-8 kit	Biolegend	Catalog# 431504
MitoSOX	Invitrogen	Catalog# M36008
MitoTEMPO	Sigma-Aldrich	Catalog # SML0737
S-Trap™ micro columns	Protifi	Catalog #C02-micro
iBlot™ 3 Transfer Stacks, mini, nitrocellulose	ThermoFisher	Catalog # IB33002

**Deposited data**		

RAW data Persulfidome	ProteomeXchange	https://doi.org/10.6019/PXD076106

**Experimental models: Cell lines**		

A549	ATCC	CCL-185
A549^CTH−/−^	EditGene	EDC07545
A549^S100A9−/-^	EditGene	EDJ0003-K03

**Oligonucleotides**		

56-FAM/AC TCA CTA TrAG GAA GAG ATG/3IABkFQ/	Integrated DNA Technologies	DS Substrate
CAT CTC TTC TCC GAG CCG GTC GAA ATA GTG AGT	Integrated DNA Technologies	17E DNAzyme (17E)

**Software and algorithms**		

FlowJo	Tree Star Inc	v10.8.2
Prism	GraphPad	v10.4.1
Proteome Discoverer software	ThermoFisher	v2.5.0.400
Sequest HT	ThermoFisher	v2.5.0.400
R, WGCNA package	R-Project	v70-4
R, DEqMS package	R-Project	v1.13.0
Gene Ontology platform	Gene Ontology	https://geneontology.org
SRPlot	WeiShengXin Shanghai Newco Bio	https://www.bioinformatics.com.cn/
Interactivenn	Heberle et al.^50^	https://www.interactivenn.net/

**Other**		

Dionex UltiMate® 3000 Rapid Separation LC (RSLC)	Thermo Fisher Scientific	N/A
QE HF mass spectrometer	Thermo Fisher Scientific	IQLAAEGAAPFALGMBDK
Amersham ImageQuant 800	Cytiva	N/A
iBlot 3 Western Blot Transfer System	Thermo Fisher Scientific	N/A

### Experimental model and study participant details

#### Aspergillus fumigatus culture

The *A. fumigatus* wild-type reference strain ATCC46645^67^ or a derived strain that constitutively expresses cytosolic GFP^68^ were used for all experiments. The fungus was routinely grown in PDA (Oxoid) at 37°C for spore harvesting.

#### Cell culture

A549 original was obtained from the company EditGene, who also constructed its derivative lines A549^CTH−/−^ and A549^S100A9−/-^. A549 was authenticated by EditGene using STR profile. The cells were not tested for mycoplasma contamination.

The cells were cultured in Dulbecco’s Modified Eagle Medium (DMEM) with high glucose, L-glutamine, sodium pyruvate and sodium bicarbonate (Sigma-Aldrich) supplemented with 10% Fetal Bovine Serum (Sigma-Aldrich) and Penicillin-Streptomycin (Sigma-Aldrich) at 37°C and 5% CO_2_.

A549 was originally isolated from a white, 58-year-old male with lung cancer. The cellular immune response and persulfidation levels may differ between males and females. Fungal infections, and in particular aspergillosis infections, are more common in males than in females We have not addressed this point, thus we acknowledge the limitations of the study.

### Method details

#### Calprotectin *in vitro* hyperoxidation and persulfidation

Recombinant human S100A8/S100A9 heterodimer, calprotectin, was purchased from Bio-techne/R&D Systems. For each condition tested, 0.25 μg calprotectin was used. Prior to its use, calprotectin was reduced with 0.1 M DTT (dithiothreitol, Sigma-Aldrich) in 50 mM HEPES (4-(2-hydroxyethyl)-1-piperazine-ethanesulfonic acid, Sigma-Aldrich) for 30 min at 25°C, followed by desalting with Spin desalting columns Zeba (Thermo-Scientific). To persulfidate calprotectin, it was incubated in 125 μM potassium (poly)-sulfide (Sigma-Aldrich) for 30 min at 37°C, followed by desalting with Spin desalting columns Zeba (Thermo-Scientific). To hyperoxidate calprotectin (previously persulfidated and not), it was incubated in 2 μM HOCl (derived from sodium hypochlorite 14% in water, Fluorochem). Oxidation was stopped with 1 mM L-Methionine (Sigma-Aldrich), and calprotectin was again desalted with Spin desalting columns Zeba (Thermo-Scientific). Finally, 1 mM CaCl_2_ (Sigma-Aldrich) was added to calprotectin (untreated, oxidized and persulfidated+oxidized) to facilitate tetramerization and calprotectin was added to the cell media according to each condition.

#### Killing assays and IL-8 measurement

The killing assay was performed as reported previously.^31^ Briefly A549, A549^CTH−/−^ or A549^S100A9−/-^ cells were seeded in 24-well plates at a concentration of 2×10^5^ cells/mL, challenged with opsonized conidia of GFP expressing *A. fumigatus* at a multiplicity of infection of 1:5 and incubated a 37°C with 5% CO_2_. The opsonization of conidia was carried out by incubating the spores in FBS at 37°C for 30 min. When specified, GYY4137 (dichloromethane-free, Cymit/Biosynth) was added at 100 μM 1 h before challenge with conidia, and 0.25 μg recombinant human S100A8/S100A9 heterodimer (biotechne/R&D Systems), untreated, oxidized or persulfidated+oxidized at the time of infection. Four and a half hours after conidia challenge, media was collected, and cells were lysed with Milli-Q water (pH 11) and sonicated to recover intracellular conidia. Finally, conidia were stained with propidium iodide (PI; 10 μg/mL, Sigma), and the percentage of dead conidia was assessed using the ratio of PI^+^ (dead conidia)/GFP^+^ (live conidia) + PI^+^ (dead conidia).

To measure IL-8 production, media collected after incubation was processed using the ELISA MAX Deluxe Set Human IL-8 kit (Biolegend) following the manufacturer’s instructions. For each experiment, the standards provided in the kit were used to plot a standard curve in GraphPad Prism (version 10.4.1), using a sigmodial 4-parameter logistic curve fitting algorithm, as described in the manufacturer’s instructions.

#### Measurement of calprotectin Zn^2+^ chelation activity

The calprotectin Zn^2+^ chelation activity was measured following a published protocol.^61^ Briefly, 0.25 μg of recombinant calprotectin were treated as described above. Different amounts of calprotectin (0, 0.35, 0.7, 1.7 or 3.4 μM) were incubated for 30 min at 25°C in a suspension of 100 mM HEPES pH 8 containing 4.2 μM of ZnCl_2_. At this point, 30 nM of DNAzyme 17E and 150 mM of DS substrate were added, and the reaction was allowed to continue for 30 min at 25°C. The reaction was stopped by adding 12 μL of EDTA 0.5 M and the fluorescence was read with an excitation of 490 nm and emission of 520 nm in a TECAN Infinite 200 plate reader. The experiment was repeated three times independently.

#### Calprotectin proteolysis assay

An amount of 1.25 μg of recombinant calprotectin per condition was treated as described above, except that CaCl_2_ was not added at the end. To each condition, 10 ng of trypsin were added, and the reactions were incubated at 37°C. Aliquots were taken at 0, 10, 30 and 90 min and run on a 15% native acrylamide gel under non-denaturing electrophoretic conditions. The gel was stained with Lumitein protein gel stain (Biotium), following the manufacturer’s instructions. The signal was recorded in an Amersham ImageQuant 800.

#### mitoROS production assay

For mitochondrial reactive oxygen species (mitoROS) assessment, A549 and A549^CTH−/−^ cells were seeded in 24-well plates at a concentration of 2×10^5^ cells/mL, incubated for 18 h prior to being challenged with Calcofluor White (CFW) labeled and opsonized conidia at a multiplicity of infection of 1:5 and incubated at 37°C with 5% CO_2_. *A. fumigatus* conidia were labeled with CFW by incubating a suspension containing 5×10^7^ conidia with 1 mg/mL of Calcofluor White (Sigma-Aldrich) in 1X PBS (Gibco) at RT for 30 min. The labeled conidia suspension was then centrifuged at 13,000 rpm for 10 min and washed with PBS to remove excess CFW. The opsonization of conidia was carried out as previously described. Four and a half hours after conidia challenge, the culture medium was removed, and cells were trypsinized for 10 min at 37°C. Cells were then centrifuged at 2,000 rpm for 5 min and subsequently resuspended in PBS containing 5 μM MitoSOX (Invitrogen; M36008), followed by 30 min incubation at 37°C, protected from light. Data were obtained on a BD FACS LSRII instrument (Becton Dickinson) and processed using FlowJo v10.8.2 (Tree Star Inc.). Mean Fluorescence Intensity (MFI) data was collected for 3 replicates per condition per experiment. The experiment was independently repeated 4 times. Due to high inter-experimental variability in the MFI values, data was normalized to the A549 value in each experiment.

For mitochondrial ROS scavenging experiments, A549 cells were pre-treated with 20 μM mitoTEMPO (Sigma-Aldrich) for 2 h prior to CFW-labeled *A. fumigatus* infection, and the compound was maintained throughout the duration of the infection. MitoSOX staining and killing assays were then performed as described previously.

#### Enrichment and analysis of persulfidated proteins

A549 or A549^CTH−/−^ cells were seeded in 24-well plates at a concentration of 10^5^ cells/mL, challenged with 10^6^ conidia (multiplicity of infection 1:10) or 100 μM of GYY4137, as described in each experimental condition and incubated for 4 h before cell disruption with RIPA buffer (Thermo Fisher Scientific). This setup was performed twice, to obtain two independent biological replicates of each condition. The detection and enrichment of persulfidated proteins was performed using the dimedone switch method.^33^ Briefly, proteins were incubated with 5 mM NBF-Cl in HEN buffer (50 mM HEPES pH 7.4, 1 mM EDTA, 0.1 mM, Neocuproine, 1% NP-40, 2% SDS) for 30 min at 37°C. The solution was then precipitated twice with methanol/chloroform (sample:MeOH:CHCl_3_, 1:1:0.25) at 4°C, 14000 × *g* for 15 min. The pellet was subsequently washed twice with methanol and resuspended in 50 mM HEPES pH 7.4 with 2% SDS. Protein concentrations of 3 mg/mL were incubated with 25 μM pre-click mix (2 mM TBTA Cu Complex, 4 mM L-arcorbic acid, 30% acetonitrile and 20 mM EDTA in PBS) containing either 0.1 mM DCP-Bio1 (for the enrichment of persulfidated proteins) or 1 mM Daz-2, 1 mM Cy5 alkyne (for in gel detection of persulfidation levels) for 1 h at 37°C, precipitated with methanol/chloroform as previously described, and resuspended in bead binding buffer (HEPES 50 mM, 1 mM EDTA, 0.5 M NaCl) or 50 mM HEPES pH 7.4 with 2% SDS, respectively.

DCP-Bio1 labeled proteins were purified with Streptavidin magnetic beads (ThermoFisher Scientific) following the manufacturer’s instructions.

#### Western blot of enriched persulfidated calprotectin

A549 and A549^CTH−/−^ cells were challenged with *A. fumigatus* conidia, as explained for the killing assay, for 1 h. Proteins were extracted and the persulfidated fraction enriched as explained above. The total and the persulfidated enriched protein fractions were run on an SDS-PAGE and transferred to a nitrocellulose membrane using the ThermoFisher iblot 3 system. The membrane was blocked in 5% skimmed milk (50 mM Tris-HCl pH 7.6, 150 mM NaCl, 0.1% Tween 20 buffer) and incubated overnight with 1:1500 Calprotectin Monoclonal Antibody (MAC387) at 4°C. The membrane was washed 5x with PBS+Tween 20 (0.1%) and incubated 1 h with 1:2000 Goat anti-mouse IgG (H + L). The membrane was washed again 5x with PBS+Tween 20 (0.1%) and subsequently incubated with SuperSignal West Pico Plus Chemiluminescence substrate (ThermoFisher Scientific) for 5 min. The signal was recorded in an Amersham ImageQuant 800.

#### Mass spectrometry

The persulfidated enriched protein fractions were reduced with 5m DTT and alkylated with 5 mM iodoacetamide (IAM) cleaned and digested using an S-Trap procedure adapted from a Protifi, which can be found at dx.doi.org/10.17504/protocols.io.yxmvmn6q9g3p/v1. Briefly, proteins were de-natured with 1.2% (v/v) phosphoric acid, loaded on the S-trap column and washed 4x with MTBE solution. Proteins were digested with trypsin 1:10 wt:wt (enzyme:protein) for 1 h at 47°C. Peptides were eluted in three consecutive steps, first with 50 mM TEAB, second with 0.1% formic acid and last with acetonitrile 30% (v/v). Subsequently samples were desalted using Corning FiltrEX desalt filter plates and POROS R3 beads. Finally, samples were concentrated using a Thermo SPD1010 speed vacuum concentrator.

Digested samples were analyzed by LC-MS/MS using an UltiMate 3000 Rapid Separation LC (RSLC, Dionex Corporation, Sunnyvale, CA) coupled to a QE HF (Thermo Fisher Scientific, Waltham, MA) mass spectrometer. Mobile phase A was 0.1% formic acid in water and mobile phase B was 0.1% formic acid in acetonitrile and the column used was a 75 mm × 250 μm i.d. 1.7 μM CSH C18, analytical column (Waters).

A 1 μL aliquot of the sample was transferred to a 5 μL loop and loaded on to the column at a flow of 300 nL/min for 5 min at 5% B. The loop was then taken out of line and the flow was reduced from 300 nL/min to 200 nL/min in 0.5 min. Peptides were separated using a gradient that went from 5% to 18% B in 34.5 min, then from 18% to 27% B in 8 min and finally from 27% B to 60% B in 1 min. The column is washed at 60% B for 3 min before re-equilibration to 5% B in 1 min. At 55 min the flow is increased to 300 nL/min until the end of the run at 60 min.

Mass spectrometry data was acquired in a data directed manner for 60 min in positive mode. Peptides were selected for fragmentation automatically by data dependant analysis on a basis of the top 12 peptides with m/z between 300 and 1750Th and a charge state of 2, 3 or 4 with a dynamic exclusion set at 15 s. The MS Resolution was set at 120,000 with an AGC target of 3e6 and a maximum fill time set at 20 ms. The MS2 Resolution was set to 30,000, with an AGC target of 2e5, a maximum fill time of 45 ms, isolation window of 1.3Th and a collision energy of 28.

Raw data was analyzed using Proteome Discoverer software (ThermoFisher, version 2.5.0.400). The data was processed using the consensus workflow provided with PD in the file CWF_Comprehensive_Enhanced Annotation_LFQ_and_Precursor_Quan and the processing workflow provided with PD in the file PWF_QE_Precursor_Quan_and_LFQ_SequestHT_Percolator. The processing workflow was set to search 703, 310 spectra against the protein database Homo sapiens (sp_canonical TaxID = 9606) (v2021-03-31) (20, 197 sequences) using the Sequest HT (version that was provided with the version of PD) software. The protein identification search engine parameters used were.

• the enzyme Trypsin (cleaving at Lysines and Arginines except where the presence of a C-terminal Proline obstructs cleavage)
• the precursor tolerance of 10 ppm and a fragmentation tolerance of 0.02 Da
• the fixed modifications of Carbamidomethyl (+57.021 Da) to Cysteine and variable modifications of Oxidation (+15.995 Da) to Methionine and Sulfide (+31.972 Da) to Aspartic Acid and Tryptophan.

A false discovery rate (FDR) is calculated for both the protein level and the peptide level by Proteome Discoverer. Proteins were labeled with High confidence where the FDR is less than 0.01; Medium confidence where the FDR is between 0.01 and 0.05; and Low confidence where the FDR is greater than 0.05. A target FDR cutoff of 0.01 was applied.

The data is normalised according to the Total Peptide Amount. It is then scaled. The raw abundances are provided in the Excel file - there are two types of Columns. The columns that start with “Abundance: F” are the raw abundance of the protein, one for each sample. The columns that start with “Abundance (Normalized): F” are the normalised values of the protein, one for each sample.

A step-by-step description of the methodology can be found in Data S1.

The mass spectrometry proteomics data have been deposited to the ProteomeXchange Consortium via the PRIDE^69^ partner repository with the dataset identifier Proteome central: PXD076106 and https://doi.org/10.6019/PXD076106.

#### Signed weighted gene correlation network analysis (WGCNA) of the persulfidome

WGCNA was performed in R using the WGCNA package (version 1.70–4).^70^ Normalized protein abundances from LC/MS were filtered to remove proteins with missing values (zero counts) in more than half of the samples, resulting in the exclusion of 170 proteins (Table S2). Samples were clustered by hierarchical clustering with average linkage based on Euclidean distances to detect outliers; based on this analysis, sample F13 (CTH^−/−^-G-A) was excluded. A soft-thresholding power (β) of 14 was selected to approximate a scale-free network. Modules were identified using a signed network with the following parameters: power = 14, minModuleSize = 15, and mergeCutHeight = 0.25. To define module-trait relationships, trait data were coded as binary variables representing cell line (A549 or CTH), presence/absence of GYY4137, and presence/absence of *A. fumigatus*. Module eigengenes were correlated with trait data using Pearson correlation with pairwise complete observations, and statistical significance was assessed using a Student’s *t* distribution.

#### Differential protein persulfidation analysis

Differential protein persulfidation analysis was performed in R using the DEqMS package (version 1.13.0).^45^ DEqMS was used to determine differentially expressed persulfidated proteins based on normalized protein intensities from label-free LC/MS. Proteins identified with high confidence (FDR <0.01) by LC/MS were retained for DEqMS analysis. For each pairwise comparison, protein intensities were log2-transformed, proteins with zero counts in one or more samples were removed, and the data were median centered. Differential expression analysis was performed using DEqMS, which applies linear modeling and Empirical Bayes variance estimation, followed by bias correction of the variance estimates via the spectraCounteBayes function.

#### Gene Ontology analyses

Analysis of enriched biological processes and KEGG pathways were performed at the Gene Ontology platform^71^^,^^72^^,^^73^ (https://geneontology.org/). Visual representations as dotplots were generated using the SRPlot free website tool^74^ (https://www.bioinformatics.com.cn).

### Quantification and statistical analysis

For WGCNA and DEqMS analyses, significance was calculated using the correspondent *R* module. For WGCNA, module eigengenes were correlated with trait data using Pearson correlation with pairwise complete observations, and statistical significance was assessed using a Student’s *t* distribution. DEqMS applies linear modeling and Empirical Bayes variance estimation, followed by bias correction of the variance estimates via the spectraCounteBayes function.

For biological data, statistical analyses were conducted using GraphPad Prism (v10.4.1). In Figure 1 (all panes), Figures 5A and 5B and Figure 6 (all panels), significance was calculated using a one-way ANOVA with Tukey’s multiple comparisons. In Figure 5C Data was analyzed using a one-sample *t* test to the hypothetical mean value of 100. Each point in the graphs depicts an independent biological replicate.

## Supplemental information

Table S1. Modules of proteins detected by the signed WGCNA analysis

Table S2. List of proteins removed from the signed WGCNA analysis (A) Proteins removed and indication of samples where they were not detected (NA). (B) Predicted function of proteins of interest

Data S1. Mass spectrometry identification of persulfidated proteins (persulfidome) (A) All mater proteins. (B) Peptide groups. (C) Combined data. (D) QC plots. (E) Methodology

Data S2. Proteins identified by differential abundance analysis by DEqMS in A549 VS A549-G+A (A) All proteins and statistical data. (B) List of proteins up- and down-persulfidated

Data S3. Proteins identified by differential abundance analysis by DEqMS in A549 VS A549+G+A (A) All proteins and statistical data. (B) List of proteins up- and down-persulfidated attending to the FDR. (C) List of proteins up- and down-persulfidated attending to the *p* value

Data S4. Proteins identified by differential abundance analysis by DEqMS in A549 VS A549+G-A (A) All proteins and statistical data. (B) List of proteins up- and down-persulfidated

Data S5. Proteins identified by differential abundance analysis by DEqMS in CTH vs. A549 (A) All proteins and statistical data for the CTH^−/−^ vs. A549^−/−^ comparison. (B) List of proteins up- and down-persulfidated in the CTH^−/−^ vs. A549^−/−^ comparison. (C) All proteins and statistical data for the CTH−/+ vs. A549−/+ comparison. (D) List of proteins up- and down-persulfidated in the CTH−/+ vs. A549−/+ comparison. (E) All proteins and statistical data for the CTH^+/−^ vs. A549^+/−^ comparison. (F) List of proteins up- and down-persulfidated in the CTH^+/−^ vs. A549^+/−^ comparison. (G) All proteins and statistical data for the CTH^+/+^ vs. A549^+/+^ comparison. (H) List of proteins up- and down-persulfidated in the CTH^+/+^ vs. A549^+/+^ comparison. (I) List of proteins that are only differentially persulfidated in the -G+A comparison
